# Treatment of HIV-2 infection: a retrospective study from a Portuguese center

**DOI:** 10.1186/1758-2652-13-S4-P54

**Published:** 2010-11-08

**Authors:** N Marques, D Seixas, P Crespo, L Malcata, J Vaz, V Mota, C Morais, J Oliveira, V Duque, J Saraiva da Cunha, A Meliço-Silvestre

**Affiliations:** 1 University Hospitals of Coimbra, Infectious Diseases, Coimbra, Portugal

## Purpose of the study

Tailoring of antiretroviral therapy (ART) in HIV-2 remains unclear, therefore therapeutic experience in this population is presented.

## Methods

Retrospective analysis of HIV-2 infected patients (pts) on ART followed at a single Portuguese center, between 1988 and 2010.

## Summary of results

37 pts were included; 19 (51%) female and 28 (76%) Caucasian. Mean ages were: 46 years [18-77] at HIV-2 diagnosis and 48 years [20-81] at the beginning of ART. The majority (54%; 20) was probably infected in West Africa through heterosexual intercourse (75%, 28). The average T CD4+ nadir was 137 cells/mm^3^ [3-586] and mean TCD4+ count prior to ART initiation was 205 cells/mm^3^. The main reason for starting ART was immunological deterioration (87%; 32) followed by pregnancy (8%; 3). Ten pts (27%) had an AIDS defining illness prior to ART initiation and 4 (11%) developed it while on ART. The mean value of the last TCD4+ count was 305/mm^3^. Overall the median number of regimens received per patient (pt) was 3 [1-7]; 12 (32%) pts took just one regimen. Mean length of ART was 61 months [1-228] and mean duration of the last prescribed regimen was 23 months [0.25-125]. Viral load ("in house" assay) was always undetectable in 19 (51%) pts. Switch of ART occurred in 21 (57%) pts with 1.4 reasons/pt. The main cause for switching was GI intolerance (57%; 12), followed by failure (38%; 8), toxicity (29%; 6) and simplification (19%; 4).

The last prescribed regimens were as follows:

a) 73% (27): 2 NRTIs + 1 PI [NRTIs: TDF/FTC (9), AZT/3TC (7), TDF/3TC (6), others (5); PIs: LPV/r (13); SQV/r (8); IDV/r (3); others (3)];

b) 19% (7): 2NRTIs (FTC/TDF) + 1 PI (DRV/r) + 1 INSTIs (RAL);

c) 8% (3): dual/triple NRTIs.

Only one pt on RAL was ART naïve. After a median follow-up of 11 months [4-24] on RAL based regimens, 3 pts had undetectable viral load (the remaining had it from the beginning). Median length of previous ART was 77 months with an average of 2.4 regimens/pt. The median gain of T CD4+ with RAL regimen was +94 cells/mm^3^ [3-239]. Currently: 21 (57%) pts are kept on follow up, 19 (90%) of whom on ART; 9 (24%)pts died (4 with AIDS related deaths) and 7 (19%)were lost to follow-up.

## Conclusions

This population had a fairly good T CD4 cell recovery while on therapy which is the best approach to assess treatment response together with clinical improvement. NRTIs/PI/INSTI containing regimens appears to be effective, although longer term outcomes are needed.

